# Zygomatic Implant Insertion in MRONJ: A Case Report with a Follow-Up of 3 Years

**DOI:** 10.3390/jcm12093300

**Published:** 2023-05-05

**Authors:** Funda Goker, Emma Grecchi, Massimo Del Fabbro, Salvatore Longoni, Luigi Vito Stefanelli, Francesco Grecchi

**Affiliations:** 1 Department of Biomedical, Surgical and Dental Sciences, University of Milano, 20122 Milano, Italy; 2 Fondazione IRCCS Ca’ Granda Ospedale Maggiore Policlinico, 20122 Milano, Italy; 3 Department of Surgery and Translational Medicine, University of Milano Bicocca, 20900 Monza, Italy; salvatorelongoni@gmail.com; 4 Private Practice, Viale Leonardo Da Vinci 256, 00145 Rome, Italy; 5 IRCCS Orthopedic Institute Galeazzi, Via Riccardo Galeazzi, 4, 20161 Milano, Italy

**Keywords:** zygomatic implants, bisphosphonates, medication-related osteonecrosis of the jaws, MRONJ, oral rehabilitation, MRONJ management

## Abstract

The oral rehabilitation of MRONJ patients represents a challenging situation. Conventional dental implant insertion is not indicated because of the risk of creating a new necrotic area. This case study describes the oral rehabilitation of a 78-year-old female patient, who developed an osteonecrotic lesion in the fourth year of bisphosphonate treatment. The patient underwent a series of surgeries, including resection of the necrotic tissue on the right maxillary region and removal of conventional implants. The patient had a large maxillary defect, and no other treatment modalities such as conventional prosthetic appliances and traditional dental implant insertions were applicable. The patient had a very poor quality of life and as a rehabilitation option, two zygomatic implant insertions were planned and performed as an anchorage for maxillary fixed prosthesis. Radiographic and clinical examination after three years of follow-up indicated that healing was achieved, and healthy tissues formed around zygomatic implants. The patient did not suffer from any additional necrotic tissues or other complications in the oral cavity. According to the results of this case report, zygomatic implantation after resective surgery might be considered as a promising alternative for MRONJ patients with large defects when other treatment alternatives fail or are not applicable.

## 1. Introduction

Currently, bisphosphonates (BP) are first-line antiresorptive agents that are widely used to treat osteoporosis, and other conditions that exhibit bone fragility. BPs act by inhibiting the resorption of trabecular bone by osteoclasts, by stimulating osteoclast apoptosis along with progressively reducing the physiological bone turnover. Additionally, they have antiangiogenic effects. As a result of medication with BPs, the bone becomes more fragile, and its repair capacity decreases [1,2]. Indications for BP therapy include osteoporosis and oncologic patients to prevent bone metastases. The most important therapeutic action of bisphosphonates is their inhibition of bone resorption which commences within 1–2 days after administration. Due to the nature of the disease, the treatment is applied for a long time. However, long-term application of BP increases the risk of bisphosphonate-associated osteonecrosis of the jaw (BRONJ) [1,2,3].

Osteonecrosis (ON), which is also called avascular necrosis or aseptic necrosis, is the death of bone cells due to decreased blood flow. In recent years, additional several cases of ONJ have been described in patients not just under BP therapy, but using other antiresorptive agents (such as denosumab) or antiangiogenic therapies. Consequently, in 2014, the American Association of Oral and Maxillofacial Surgeons (AAOMS) recommended to change the initial definition of BRONJ to medication-related osteonecrosis of the jaw (MRONJ) [4].

Currently, MRONJ is defined as a rare and severe adverse drug side effect that may occur in cancer and osteoporosis patients being treated with antiresorptive and/or antiangiogenic medications. It is a progressive bone destruction in the maxillofacial region, which can be accompanied by pain, paresthesia, swelling, soft tissue ulceration, suppuration, and intra/extra oral sinus tracts [4,5]. Oral rehabilitation with conventional dental implant placement is not indicated in MRONJ patients, due to the risk of creating a new necrotic area. In such patients, functions such as chewing, speech, and thus quality of life are considerably reduced. Moreover, in the majority of these patients, treatment is quite demanding and mostly not possible [1].

The mandible and maxilla are the most common regions of MRONJ [6]. Although the etiology of MRONJ has not yet been fully explained, the most widely accepted theory is that the jawbone shows increased bone turnover compared to other skeletal regions. Therefore, it is more frequently affected by bone-turnover-modifying agents, such as bisphosphonates [3,7,8]. An alternative bone site for the anchorage of implants for fixation of dental prostheses, such as the zygomatic region, might be an alternative in such patients for the enhancement of quality of life in terms of function.

Currently, zygomatic implant insertion in patients with severely atrophic maxilla for oral rehabilitation is considered as a successful treatment modality in patients with edentulous atrophic maxilla for improved function and esthetics [9,10,11,12]. However, zygomatic implant placement is to this day debated in patients that use bisphosphonates or with a history of such medications [13].

The aim of this case report was to evaluate the outcomes of oral rehabilitation using zygomatic implants as anchorage for fixed maxillary prosthesis for a MRONJ patient that had a residual large defect following resection surgery. The patient presented in this study previously had a series of surgical interventions including dental implant removals and surgical debridement of the necrotic tissue and had a large maxillary defect which compromised the quality of life of the patient. The patient underwent an additional surgery, and two zygomatic implants were inserted as an anchorage for maxillary fixed prosthesis.

## 2. Clinical Report

This case study describes the oral rehabilitation of a 78-year-old female Stage 2 MRONJ patient with zygomatic implants. The treatment presented was carried out in accordance with The Code of Ethics of the World Medical Association (Declaration of Helsinki) for medical research. A signed informed consent agreement form was obtained from the patient for the intervention and therapy. An additional voluntary written consent form was retrieved from the patient for using her data and images for scientific purposes.

### 2.1. Medical Anamnesis of the Patient

The patient was under treatment for osteoporosis since January 2012 with 70 mg Doryx© (Alendronic acid) antiresorptive bisphosphonate oral tablets taken once a week (Doppel Farmaceutici Srl, Rozzano, Italy) and Eurocal D3© Calcium + Vitamin D3 tablets (calcium carbonate 2500 mg/cholecalciferol 880 U.I.) (Amdipharm Ltd., Dublin, Ireland) taken once a day. The initial symptoms of MRONJ developed about 3 and half years after she started bisphosphonate medication (in April 2016).

The patient was also under hypertension therapy with Blopresid© 8 mg/12.5 mg tablets taken once a day (candesartan cilexetil and hydrochlorothiazide—an angiotensin II receptor (type AT) antagonist and diuretic, respectively) (Takeda Italia SpA, Cerano, Italy). The patient was otherwise healthy without any other major systemic diseases.

### 2.2. Oral Rehabilitation History of the Patient

**Figure 1:**
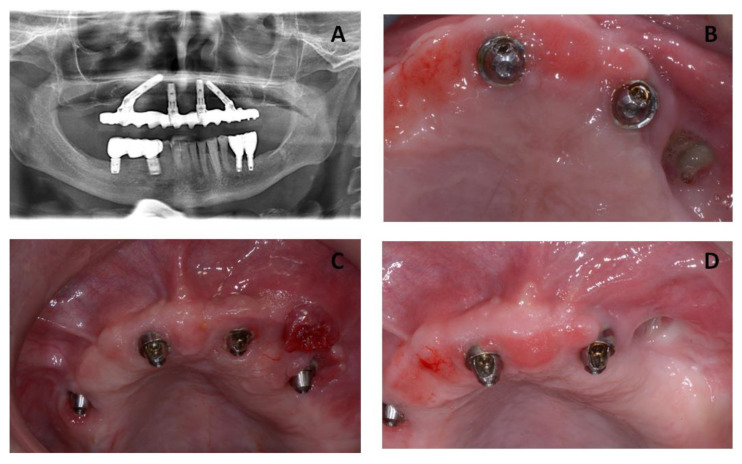
(**A**) Radiographic image from the patient showing an MRONJ lesion on the posterior left side of the maxillary bone. (**B**–**D**) Clinical images from the patient showing an MRONJ lesion on the posterior left side of the maxillary bone.

Initial visits of the patient in 2014. Maxillary site: The patient had an edentulous maxilla with two conventional implants in the region of #22 and #26 (tilted implant) and #12 and #16 (tilted implant) which were inserted in February 2014 as an anchorage for All-On-4 bridge in November 2014. Mandibular site: The patient also presented a partially edentulous lower jaw with seven natural teeth (#34, #33, #32, #31, #41, #42, and #43), and four conventional implants (#44, #46, #35, and #36) which were inserted in 2007, supporting two bilateral fixed bridge prostheses that were delivered for the renewal of prostheses for esthetic and functional reasons and for better occlusion in November 2014 (Figure 1).

April 2016: The patient reported problems consisting of poor chewing function. After clinical and radiographic examination, signs of osteonecrotic lesion on her left maxillary bone and right mandible were diagnosed. In the clinical examination, the patient presented exposed and necrotic bone that could be probed to the bone, associated with pain and erythema in the region of exposed bone with purulent drainage.

Treatment was planned with a suspension of bisphosphonate oral tablets and removal of the osteonecrotic bone. The pre-operative surgical protocol included a prescription of antibiotics (Augmentin 1 gr) for one week starting from one day before the surgery. A week before surgery, a professional oral hygiene session was given with a prescription of chlorhexidine digluconate 0.2% mouthwash. The elimination of bone sequestration (superficial debridement) was planned, as a primary step in the healing process, and was accomplished with surgical procedures that consisted of removing necrotic and damaged tissues, which could otherwise compromise the healing of the lesion.

The patient underwent operations for dental implant removals and surgical debridement of the necrotic tissue which might compromise wound healing under local anesthesia in a dental clinical environment using sterile surgical instruments such as scissors, forceps, and scalpel. The zygomatic implant insertions were performed using an extra-maxillary protocol under general anesthesia. The details of the protocol used in zygomatic surgery can be found in a recent publication by the same team of authors [10].

**Figure 2:**
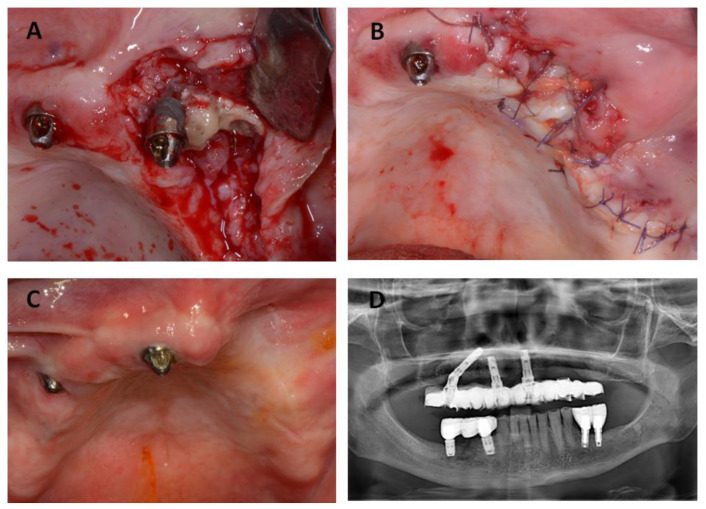
(**A**,**B**) Intra-operative image from the patient showing removal of the maxillary dental implants and osteonecrotic lesion. (**C**) Intra-oral post-operative view of the patient after a month showing healthy tissues. (**D**) The radiographic image shows the post-operative situation with the previous prosthesis adjusted to the new situation.

The patient underwent three surgeries in total. The first surgery was scheduled for September 2016 and included removal of the necrotic bone and implant #26 (Figure 2). After a healing period of three months, the All-on-4 prosthesis was placed for function and esthetics.

**Figure 3:**
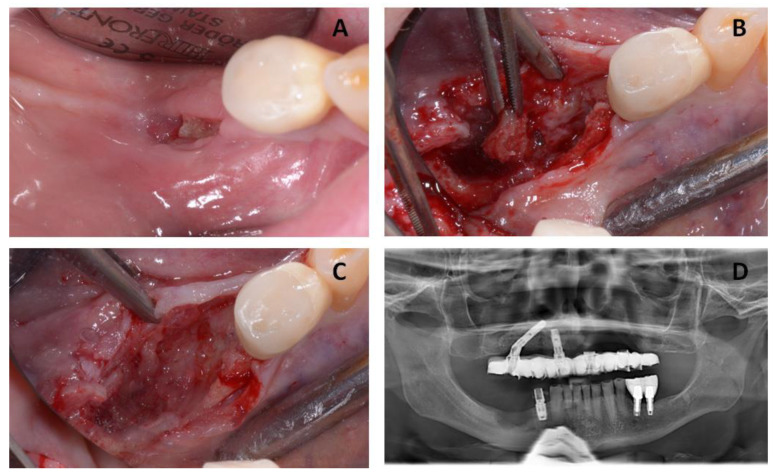
(**A**) Clinical images from the patient showing a new MRONJ lesion on the posterior right side of the mandibular bone. (**B**,**C**) Intra-operative image from the patient showing the removal of the sequester. (**D**) The radiographic image shows the post-operative situation.

The second surgical session in October 2016 included the removal of implant #46 and the osteonecrosis lesion on the posterior right side of the mandibular bone (Figure 3).

February 2017: The patient received additional surgery that included the debridement of the bone sequestrum and removal of the implant at #21. After removal of the anterior dental implant, the patient did not receive any fixed maxillary prosthesis. At this point, a removable provisional prosthesis with soft lining material was delivered to monitor the healing period. A fixed prosthesis was planned, with two additional zygomatic implants as anchorage, in case of complete relief from the symptoms of MRONJ.

**Figure 4:**
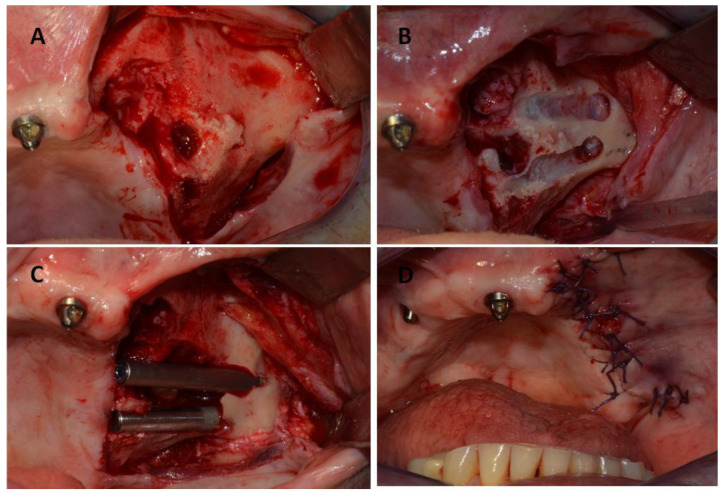
(**A**,**B**) Intra-operative images of the patient showing the steps of two zygomatic implant preparations on the posterior left side of the maxillary bone. (**C**) Insertion of two zygomatic implants. (**D**) Intra-operative image showing the mucoperiosteal flap being repositioned and sutured.

After total healing was achieved with no complications and no further signs of relapse of the MRONJ lesion, two zygomatic implants were inserted in May 2017. The zygomatic implants used in this study were Zygomatic Noris ^®^ (Noris Medical Ltd., Nesher, Israel) with sizes of 45 mm (REF: NM-F4445) and 35 mm (REF: NM-F4435) in length and 3.5/4.2 mm in diameter, with 45° angled multi-unit abutments (Figure 4).

**Figure 5:**
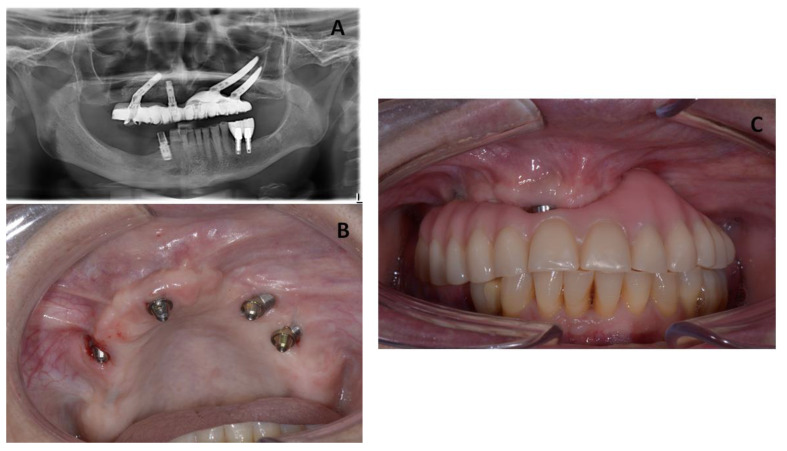
(**A**) Radiographic images from the patient showing healing of the MRONJ lesions with two zygomatic implants inserted after one month of follow-up. (**B**) Clinical images from the patient showing healing of the MRONJ lesions with two zygomatic implants inserted. (**C**) Intra-oral view showing the final prosthesis.

In the first follow-up appointment after one month, the patient exhibited healthy oral tissues with no signs of complications. A final Toronto prosthesis was delivered at six months after implant insertion (Figure 5).

**Figure 6:**
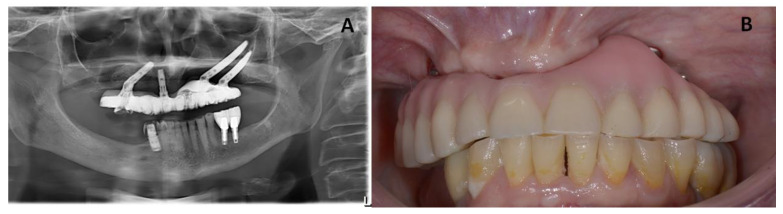
(**A**) Panoramic radiograph of the patient after 3 years of follow-up. (**B**) Clinical image from the patient showing occlusion.

The patient was recalled for clinical follow-up every 3 months for the first year, and then twice a year. In these appointments, any changes or complications and any adverse events were evaluated, and the occlusion was examined carefully. Additionally, the Toronto prosthesis was unscrewed to check the status of the soft and hard tissues. Figure 6 presents clinical and radiographic images taken at 3 years of follow-up, showing healthy peri-implant tissues.

## 3. Discussion

The management of MRONJ patients, especially those with large defects, is a very challenging situation both for the surgeon and the patient. There is no gold-standard therapy for MRONJ patients, and treatment strategies are mostly focused on the stage of the MRONJ lesion. Commonly accepted treatment goals include the prevention of MRONJ progression, patient education, control of pain, and managing infection, while supporting continued oncologic treatment in patients at risk [4,5]. Prophylactic dentistry before starting medication is a critical factor. Good oral hygiene status must be maintained, and restorative dental treatment and the extraction of non-restorable dentition must be performed before starting medications with the risk of osteonecrosis [4,5].

MRONJ lesions are characterized by an avascular necrotic bone area in the maxillofacial region that does not heal within eight weeks in a patient treated with an antiresorptive or an antiangiogenic agent and with no history of oral cancer or radiation therapy to the craniofacial region [14,15]. The management at the initial stages (Stage 0, Stage 1) mainly concentrates on non-invasive protocols, such as the use of pain medication, antibacterial mouth rinse, antibiotics, and clinical follow-up on a quarterly basis. In cases of advanced stages, such as Stage 2 and Stage 3, surgical debridement or resection to relieve soft tissue irritation and infection control are additional important therapies for management of the osteonecrotic lesion [4].

Generally, surgical interventions are considered as a major triggering risk factor for patients that are under treatment or have a history of medication with antiresorptive or antiangiogenic agents [4,5]. Furthermore, patients with established MRONJ should avoid additional dentoalveolar surgical procedures and dental implant insertions, to avoid additional sites that may result in areas of exposed necrotic bone. Surgical operations are advised as more appropriate for patients at advanced stages and should be avoided whenever possible [4,5]. Various treatment protocols and adjunctive therapies have been proposed in the scientific literature. Following complete removal of the necrotic tissue, the closure of the defect can be obtained using a diversity of autogenous flaps such as the mucoperiosteal flap, buccal fat pad flap, microvascular free flaps, mylohyoid flap, local myofascial, nasolabial flap, or obturator prosthesis [5].

As mentioned above, there is a great heterogeneity of surgical options; furthermore, there are several adjunctive therapy options that have been successfully utilized in dental clinics to enhance bone and soft tissue healing with different surgical procedures [5]. According to the literature, in advanced stages, most of the researchers report better MRONJ defect resolution in patients that had surgical interventions compared with patients that were treated with more conservative protocols [5]. As adjunctive options, different biological agents, such as autologous platelet concentrates, recombinant growth and differentiation factors and parathyroid hormones have been described. Additionally, laser therapy, hyperbaric oxygen therapy, ozone therapy and angiogenic drugs were reported [5].

In MRONJ patients, open, non-healing bone lesions significantly reduce the quality of life of such patients and make oral rehabilitation almost impossible, including dental implant applications [1,16]. Some of the patients can present an additional issue: even though they might have complete resolution following successful therapy, the large defect in the mandible or maxillary bone can create a very critical situation for an oral rehabilitation in terms of function and esthetics. In such cases, implants anchored in the zygomatic bone may be a valuable alternative for anchorage, to enhance the oral rehabilitation of these MRONJ patients, especially in cases when other treatment alternatives (such as conventional dental implants) fail or are not applicable.

The insertion of zygomatic implants was evaluated by various authors and several advantages were reported, such as lower costs and reduced time due to fewer surgical steps without any need for additional bone grafts [12,17]. However, the placement of zygomatic implants in a patient with a history of MRONJ is a somewhat taboo topic, since it is considered a very challenging, risky and aggressive surgery itself. The purpose of this case report was to investigate the possibility of implants to be placed in the zygomatic bone in a MRONJ patient as a treatment alternative in such patients with severe defects and compromised quality of life.

This case report describes the management of a 78-year-old female MRONJ patient with a fixed prosthesis. The patient previously had a large amount of osteonecrotic bone due to bisphosphonate intake and received oral rehabilitation with implants that had anchorage in the zygomatic bone. No other treatment modalities, such as conventional prosthetic appliances and traditional dental implant insertions, were applicable. Radiographic and clinical examination after 3 years of follow-up indicated that a complete healing of the soft and hard tissues was achieved with no recurrence of osteonecrosis and no further complications in the oral cavity. However, even though a successful result was obtained in this case report, the selection of the patient is a crucial factor. Firstly, complete healing of the MRONJ lesion must be obtained. According to the opinion of the authors of this work, the candidate patient must be otherwise healthy to be able to overcome the aggressive surgery and the surgeon must be highly experienced with the zygomatic implant surgery. The decision must be taken together with the patient with discussion of other alternatives and the mention of the possible advantages, disadvantages and risks.

## 4. Conclusions

In the literature, there are no reports on zygomatic implant insertions in MRONJ patients, and it would be impossible to reach a conclusion with one case. However, according to the results of this single case report, zygomatic implant insertion may be a feasible and promising alternative protocol for MRONJ patients with large defects at the posterior maxillary region after resection surgery. A prosthesis anchored on zygomatic implants might enhance the quality of life of the patient in a short time in terms of esthetics and function, but the risks of this procedure should be approached with caution.

## Data Availability

The authors will provide data upon request.

## Abbreviations

BP: bisphosphonates; BRONJ: bisphosphonate-associated osteonecrosis of the jaw; MRONJ: medication-related osteonecrosis of the jaw; AFF: atypical femoral fracture.
